# Emergency percutaneous needle decompression for tension pneumoperitoneum

**DOI:** 10.1186/1471-230X-11-48

**Published:** 2011-05-05

**Authors:** Costanza Chiapponi, Urban Stocker, Markus Körner, Roland Ladurner

**Affiliations:** 1 Department of Surgery, Hospital of the Ludwig-Maximilians-University, Nussbaumstr. 20, 80336 Munich, Germany; 2 Department of Clinical Radiology, Hospital of the Ludwig-Maximilians-University, Ziemssenstr. 1, 80336 Munich, Germany

## Abstract

**Background:**

Tension pneumoperitoneum as a complication of iatrogenic bowel perforation during endoscopy is a dramatic condition in which intraperitoneal air under pressure causes hemodynamic and ventilatory compromise. Like tension pneumothorax, urgent intervention is required. Immediate surgical decompression though is not always possible due to the limitations of the preclinical management and sometimes to capacity constraints of medical staff and equipment in the clinic.

**Methods:**

This is a retrospective analysis of cases of pneumoperitoneum and tension pneumoperitoneum due to iatrogenic bowel perforation. All patients admitted to our surgical department between January 2005 and October 2010 were included. Tension pneumoperitoneum was diagnosed in those patients presenting signs of hemodynamic and ventilatory compromise in addition to abdominal distension.

**Results:**

Between January 2005 and October 2010 eleven patients with iatrogenic bowel perforation were admitted to our surgical department. The mean time between perforation and admission was 36 ± 14 hrs (range 30 min - 130 hrs), between ER admission and begin of the operation 3 hrs and 15 min ± 47 min (range 60 min - 9 hrs). Three out of eleven patients had clinical signs of tension pneumoperitoneum. In those patients emergency percutaneous needle decompression was performed with a 16G venous catheter. This improved significantly the patients' condition (stabilization of vital signs, reducing jugular vein congestion), bridging the time to the start of the operation.

**Conclusions:**

Hemodynamical and respiratory compromise in addition to abdominal distension shortly after endoscopy are strongly suggestive of tension pneumoperitoneum due to iatrogenic bowel perforation. This is a rare but life threatening condition and it can be managed in a preclinical and clinical setting with emergency percutaneous needle decompression like tension pneumothorax. Emergency percutaneous decompression is no definitive treatment, only a method to bridge the time gap to definitive surgical repair.

## Background

The term pneumoperitoneum refers to the presence of air within the peritoneal cavity. The most common causes are perforated ulcers, tumours or traumas. Iatrogenic causes are leaking anastomoses, misplaced thoracenteses or pleural drains, percutaneous needle biopsies, peritoneal catheter placements, peritoneal dialysis, paracenteses, instrumental perforations of uterus or vagina, ruptured urinary bladder, perforating foreign bodies and application of compressed air and overdistension with gas during endoscopy. Perforation as a complication of upper endoscopy and colonoscopy is estimated to occur in less than 1% of procedures [1-4]. Endoscopic interventions associated with an increased perforation risk include polypectomy for polyps larger than 20 mm [5] or endoscopic mucosal resection and endoscopic submucosal dissection for colorectal neoplasia [6,7]. Patients over 75 years of age also have an approximately 4-6 fold rise in the colon perforation rate as opposed to younger patients [8-10], due to reduced colonic wall mechanical strength, as the increased rate of colonic diverticular diseases in these patients also suggests. Other risk factors for perforation reported in the literature beside diverticular disease include previous intra-abdominal surgery [1], colonic obstruction as an indication for colonoscopy [11], and female gender [12]. This seems to be due to the greater colonic length and a more mobile transverse colon in women [13].

The most common clinical feature of perforation is the visualization of an extra-intestinal structure during the endoscopic examination [4]. Some patients complain about intense abdominal pain and tenderness during or immediately after endoscopy, some present within several hours after perforation.

In patients in acute distress, complaining of dyspnea in addition to abdominal pain and fullness tension pneumoperitoneum should be suspected. They may also report shoulder pain from referred diaphragmatic irritation as after laparoscopy. On the physical exam the abdomen is usually tympanitic and rigid. Rectal prolapse or crepitus from trapping of subcutaneous air in the abdominal wall may be also seen. The vena cava can be compressed by the intraabdominal air and can result in hypovolemic shock due to decreased venous blood return to the heart.

In hemodynamically unstable patients no delay in therapy and immediate abdomen decompression should follow.

In hemodynamically stable patients radiologic studies to confirm the diagnosis of pneumoperitoneum may include a plain roentgenogram of the abdomen, sonography and computed tomography. Signs of a large pneumoperitoneum in plain supine abdominal radiography include the football sign (the intraperitoneal outlines the abdominal cavity, the falciform ligament appears like the laces of a football), the double-wall sign (the visualization of the outer wall of bowel loops caused by the presence of extraluminal and intraluminal gas) and the cupola sign (saddlebag or moustache sign, due to gas trapped under the central tendon of the diaphragma). In addition when a large pneumoperitoneum occurs, air may outline the urachus, as a thin midline linear structure in the lower abdomen from the dome of the urinary bladder or the lateral umbilical ligaments, as an inverted V in the pelvis. On a left lateral decubitus radiograph, free air usually appears around the inferior edge of the liver.

The free air is observed in sonography as an echogenic line with a posterior ring-down or reverberation artefact [14,15].

**Figure 1:**
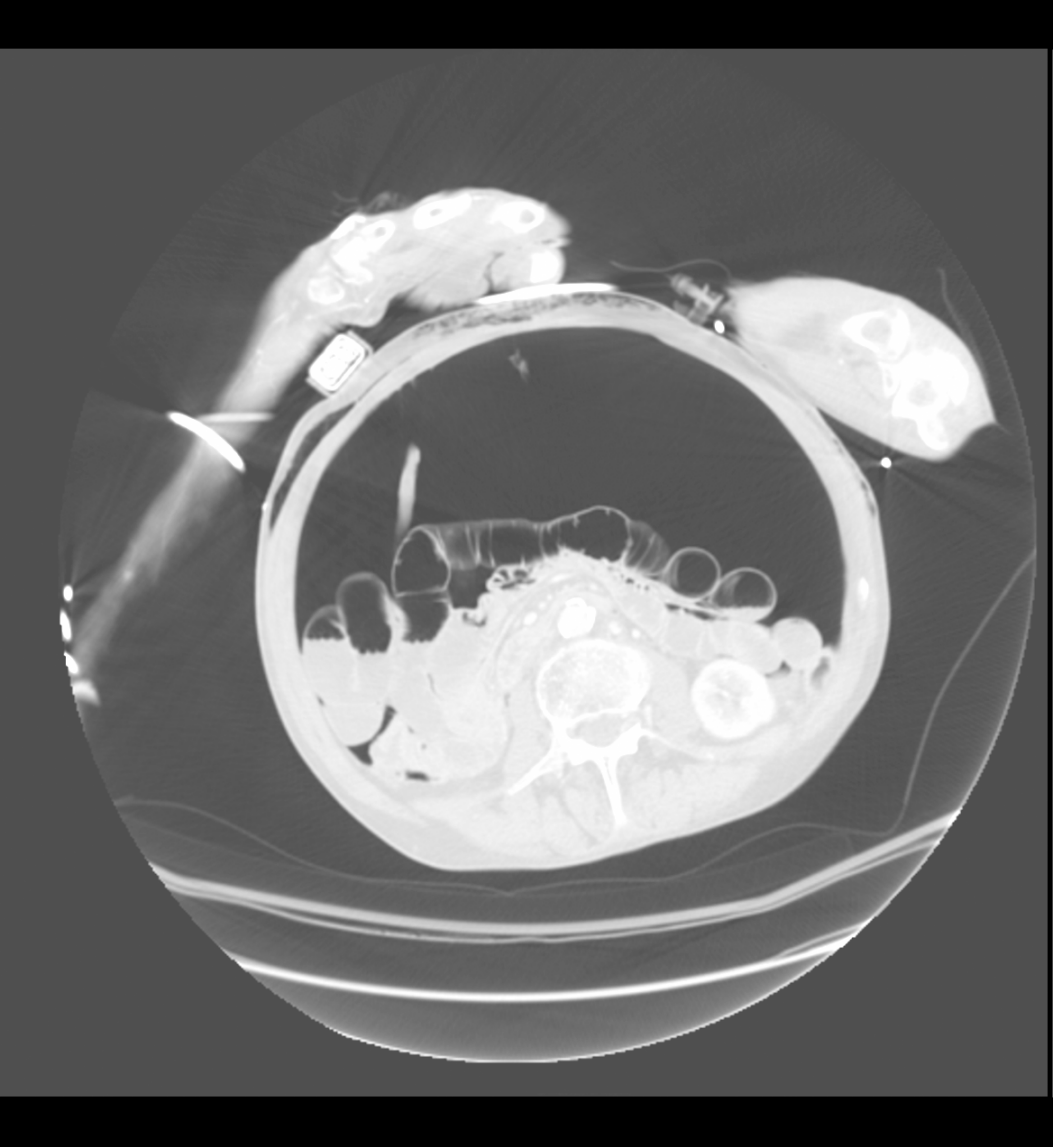
**CT scan image of pneumoperitoneum**. Axial view.

**Figure 2:**
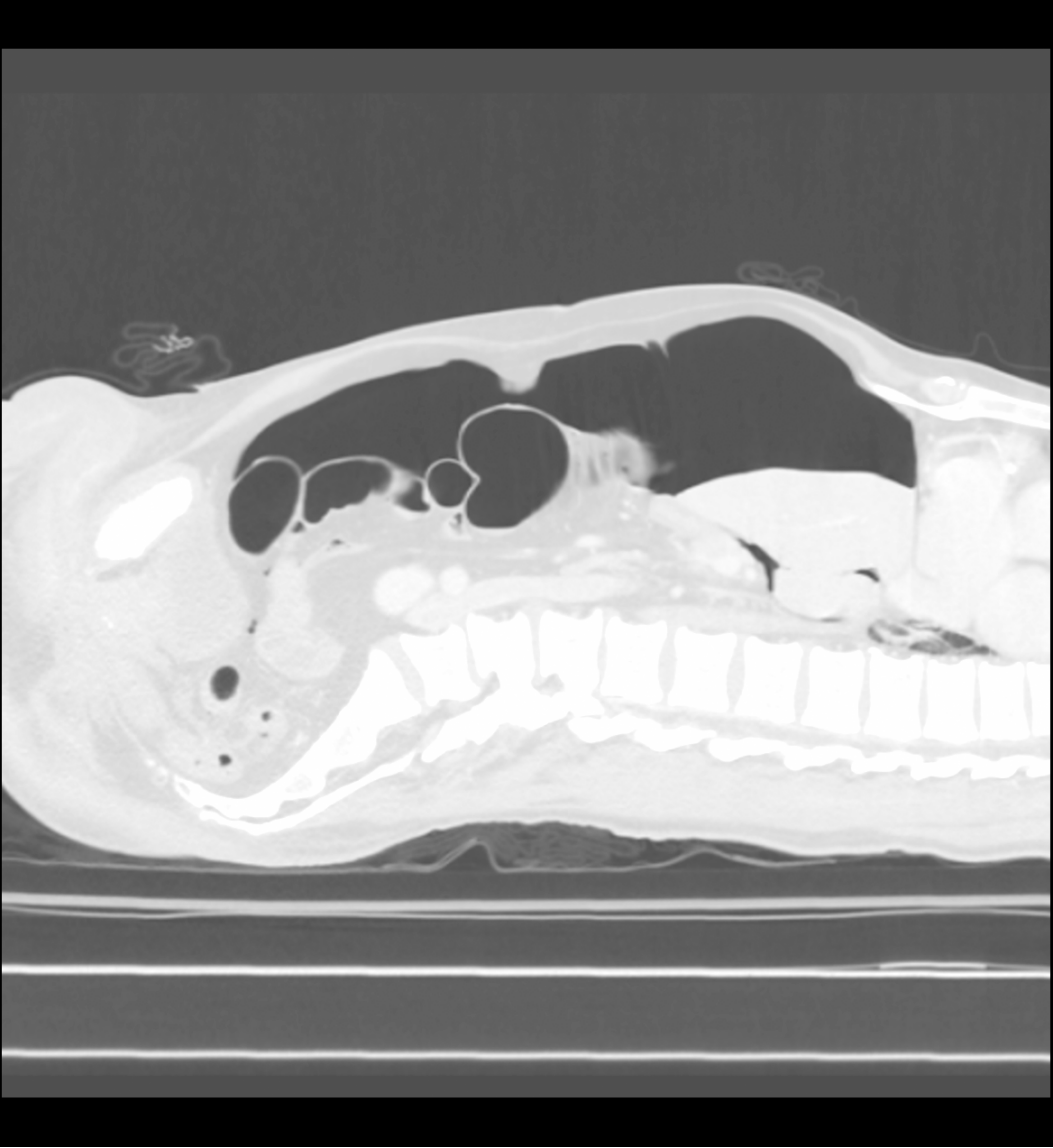
**CT scan image of pneumoperitoneum**. Sagittal view.

In a CT-scan tension peritoneum may appear as follows (Figure 1 and 2).

Once pneumoperitoneum has been diagnosed therapy should follow quickly. Less than 20% of the patients can be managed with a non-surgical approach [4,16]. This should be reserved for patients with small amount of intraperitoneal air, in good general condition and without any sign of peritonitis. Medical treatment has good results for smaller perforations, like those resulting from therapeutic colonoscopies (e.g. polypectomy), as opposed to those caused by diagnostic colonoscopies [17]. These patients should receive intravenous fluids, keep absolute bowel rest and they should be treated with intravenous broad-spectrum antibiotics. If the conservative treatment is successful, the patient's general condition should improve within one or two days. If this is not the case further investigation is due and it may be necessary to proceed to surgical management. The overall success rate of conservative management of colonic perforation varies from 33% to 73% [10].

For symptomatic patients with significant amount of free air laparotomy represents the standard surgical approach. However there is growing evidence that endoluminal repair or laparoscopic surgery can also be used to manage this condition with good or even better results [18,19]. Simple closure is possible in case of small perforations, without significant fecal contamination. Bowel resection including the perforation site is necessary in case of large perforations, or when primary closure could compromise the lumen. In the absence of significant intra-abdominal contamination, bowel resection and anastomosis can be performed with acceptable morbidity. In case of extensive inflammation or peritonitis, bowel resection with fecal diversion should be considered, due to the risk of anastomosis insufficiency. This is often the case in patients presenting 24 h after perforation.

## Methods

This is a retrospective analysis of cases of pneumoperitoneum and tension pneumoperitoneum due to iatrogenic bowel perforation during endoscopy. All patients admitted to our surgical department between January 2005 and October 2010 were included in this study.

Tension pneumoperitoneum was diagnosed in patients with

1. history of endoscopy in the last 24 hrs before presentation

2. abdominal distension and fullness

3. dyspnea

4. hypotension

5. jugular vein congestion

6. no clinical signs of pneumothorax

Percutaneous needle decompression was performed with a 16G venous catheter. This was positioned two centimetres below the umbilicus in the midline.

## Results

Between January 2005 and October 2010 eleven patients with iatrogenic bowel perforation were admitted to our surgical department. The patients mean age was 56 ± 4 years (range 35 - 86 years). Six of them were male, five were female and their mean ASA score was 2 ± 1 points. None of them had had any intestinal operation to that point. They all complained of abdominal pain indicated between 4 and 10/10 on a numeric pain scale. Three of them had clinical signs of tension pneumoperitoneum including vital compromise (dyspnea, hypotension and congested jugular veins) in addition to abdominal pain and distension. One of them had lost consciousness shortly after the endoscopy in the practice of her gastroenterologist.

All patients with suspect of pneumoperitoneum received a computed tomography of the abdomen, in which the diagnosis and even the perforation site were evident. Only in one case the radiologist could not identify the site. The perforation was found in the colon sigmoideum in 6 of 11 cases, in the coecum in two cases, in the colon transversum in two more cases and in the rectum in one case. One of the patients with a perforation in the colon transversum also had a tumour of this intestinal segment as complicating factor.

The mean time between perforation and admission was 36 ± 14 hrs (range 30 min - 130 hrs), between ER admission and begin of the operation was 3 hours and 15 minutes ± 47 minutes (range 60 min - 9 hrs) but no longer than 3 hrs in patients with evidence of tension pneumoperitoneum (Table 1). In these cases (3 of 11) emergency percutaneous needle decompression with a 16G venous catheter was successfully performed to bridge the time to laparotomy. This improved significantly the patients' condition (stabilization of vital signs, reducing jugular vein congestion).

**Table 1:** Data of patients diagnosed with intestinal perforation following endoscopy at our surgical department between October 2005 and October 2010.

number of patients	11
sex ratio (male : female)	6 : 5
age (years)	56 ± 4
ASA score	2 ± 1
Time between perforation and ER admission	36 ± 14 hrs
Time between ER admission and begin of operation	3 hrs and 15 min ± 47 min
Clinical signs of tension pneumoperitoneum	3 : 11

All patients were operated except one 35 years old patient with no comorbidities, who was treated conservatively. In this patient a biopsy in the colon transversum had been performed, causing the perforation. He presented 24 hours after colonoscopy, complaining of abdominal discomfort. He denied dyspnea, he was not hypotensive and had no congestion signs so that tension pneumoperitoneum could be ruled out. The CT-scan of the abdomen showed only a small amount of free air. He was admitted to the hospital with bowel rest and broad spectrum antibiotics. Both measures were kept over eight days. After eight days computed tomography was repeated. The perforation site was still clearly visible but the surrounding infection had decreased and there was no free air. The patient was discharged in good general condition.

The other ten patients underwent surgical repair. Nine out of ten received laparotomy, one of them laparoscopy. In 40% of all cases primary repair was possible. In 30% of the cases a bowel resection including the perforation site was necessary. In the remaining 30% of the cases signs of fecal peritonitis were visible and stoma formation was imperative (Table 2).

**Table 2:** Management of patients diagnosed with intestinal perforation following endoscopy at our surgical department between October 2005 and October 2010.

Conservative management	1 : 11
Laparoscopic approach	1 : 10
Primary repair	4 : 10
Intestinal resection with anastomosis	3 : 10
Intestinal resection with fecal diversion	3 : 10

## Discussion

The retrospective analysis of the cases of iatrogenic pneumoperitoneum following endoscopy admitted to our surgical department during the last five years showed that the time between perforation and admission was quite long (36 ± 14 hrs). All patients complained of abdominal distension and fullness. The additional presence of a tympanitic, rigid abdomen, hypotension, dyspnea, and jugular vein congestion were considered as signs of tension pneumoperitoneum, requiring immediate management. This was seen in three of the eleven cases. Since the time range between ER admission and begin of the operation was generally longer than 3 hours, in those cases emergency percutaneous needle decompression with a 16G cannula was successfully performed. This improved significantly the cardiopulmonary condition of the patients awaiting definitive surgical repair. For this reason we would like to suggest the placement of an emergency percutaneous decompression needle into the abdomen if tension pneumoperitoneum is clinically manifest in order to ease the patient's symptoms in a preclinical and clinical setting until definitive surgical repair can be performed.

This procedure is certainly insufficient to drain adequately a tension pneumoperitoneum but it helps to reduce the intra-abdominal pressure, which might cause an abdominal compartment syndrome. If proper technique is applied, it is an easy and inexpensive procedure and helps to improve the patient's condition while gaining time to organize definitive surgical treatment.

The two recommended areas of abdominal wall entry are the same as for paracentesis: two centimetres below the umbilicus in the midline (through the linea alba) and five centimetres superior and medial to the anterior superior iliac spines on either side.

To minimize complications, areas of prominent veins (caput medusa), infected skin, or scar tissue should be avoided. Ultrasonography may be used to check the selected entry site but will not be necessary in most cases.

Possible complications of percutaneous abdominal decompression include hemorrhage and bowel perforation if bowel distension and adhesion are present. However, colon or organ perforation is improbable in supine patients with amounts of intra-abdominal gas large enough to cause a tension pneumoperitoneum, as in figures 1 and 2.

The cannulas used for decompression should be thick and long enough to perforate the abdominal wall and drain enough air out of the peritoneal cavity (<18G). The body mass of the patient should also be taken into account for the choice of an adequate needle. Although thinner needles (19-23G), like those commonly used for transgastric or transintestinal CT-guided biopsies, have a lower complication rate, they are not sufficient to drain an adequate amount of air. 16G cannulas proved to be sufficient to immediately drain the trapped air in the emergency pneumothorax decompression. On the other hand they are not big enough to cause a large bowel perforation. For this reason they were used in this study.

The decompression should always be followed by a complete laparoscopic/laparotomic bowel exploration and definitive surgical repair.

To our knowledge this procedure has only been described in singular case studies so far. Here we suggest the standard use of this method for preoperative management of tension pneumoperitoneum, in order to gain time for organizing definitive repair.

## Conclusions

The procedure described in this study was successfully performed in three cases and it helped to improve the general conditions of three patients with signs of tension pneumoperitoneum, bridging the time gap to definitive surgical treatment. Also if this is a small number of cases, we would like to suggest emergency percutaneous needle decompression for the acute management of tension pneumoperitoneum like generally accepted for the acute management of tension pneumothorax. Patients with history of recent endoscopy (<24 h), presenting with abdominal distension, dyspnea, hypotension and jugular vein congestion are highly suggestive of tension pneumoperitoneum. If pneumothorax can be ruled out and pneumoperitoneum is suspected as cause of the symptoms, emergency needle decompression should be performed, as described above. This improves the patient's condition providing time to organize definitive surgical repair.

## Competing interests

The authors declare that they have no competing interests.

## Authors' contributions

CC and RL conceived this study, CC and US acquired and interpreted the data, CC and RL wrote the manuscript, MK chose the CT-scan images and revised the manuscript critically; all authors have given final approval of the version to be published.

## Pre-publication history

The pre-publication history for this paper can be accessed here:

http://www.biomedcentral.com/1471-230X/11/48/prepub
